# Surgical management of effusive constrictive pericarditis

**DOI:** 10.5830/CVJA-2013-042

**Published:** 2013-10

**Authors:** Fuat Buyukbayrak, Eray Aksoy, Serpil Tas, Kaan Kirali

**Affiliations:** Kartal Kosuyolu Heart and Research Hospital, Istanbul, Turkey; Kartal Kosuyolu Heart and Research Hospital, Istanbul, Turkey; Kartal Kosuyolu Heart and Research Hospital, Istanbul, Turkey; Kartal Kosuyolu Heart and Research Hospital, Istanbul, Turkey

**Keywords:** effusive constrictive pericarditis, surgery, pericardiectomy

## Abstract

**Background:**

The surgical approach for effusive constrictive pericarditis (ECP) has not been extensively studied. We present our institution’s early and long-term results of pericardiectomy in our cohort of patients with ECP.

**Methods:**

Diagnosis was made primarily by echocardiography. Right heart catheterisation was performed in eight patients. Pre-operatively, 10 patients had undergone at least one previous attempt at therapeutic pericardiocentesis. Pericardiectomy was performed where appropriate (thickened or inflamed).

**Results:**

Of our 12 patients (50% male, median age 48 years, range 17–72 years), the underlying aetiology included idiopathic in five (41.6%), tuberculosis in four (33%), and malignancy in three patients (25%). Elective surgery was performed in nine patients. Median values of both central venous pressure and pulmonary capillary wedge pressure decreased markedly postoperatively (from 16.5 to 11.0 mmHg, *p* = 0.02; 20.0–15.0 mmHg, *p* = 0.01, respectively). There was no in-hospital mortality. Follow up ranged from three months to nine years (median three years). Five (41.6%) patients died during the follow-up period, and cumulative two-year survival was 55.6 ± 1.5%.

**Conclusion:**

Pericardiectomy for ECP was effective, in terms of our early results, in patients unresponsive to medical therapy. Long-term survival depends on the underlying disease.

## Abstract

Effusive constrictive pericarditis (ECP) is a distinct entity in which diastolic filling is compromised by two combined mechanisms. First, increased intrapericardial pressure adversely affects volumetric expansion by causing external chamber compression. Second, loss of epicardial elasticity precludes myocardial relaxation.

ECP is defined, on the basis of simultaneous catheterisation and pericardiocentesis findings, as the persistence of high intracardiac pressures despite percutaneous drainage of the pericardial fluid. The definition should not be misinterpreted, as ECP differs from CP only in being accompanied by fluid collection around the heart. Rather, what makes ECP a distinct entity is the involvement of the visceral pericardium. The fluid collection is a consequence of the underlying inflammatory process, and may directly relate to treatment outcomes.^1^,^2^

The prevalence of ECP ranges from 1.4–14.8%,^3^ and as high as 24% in patients requiring pericardiectomy.^4^ Although ECP has commonly been linked with tuberculosis, numerous case reports associate it with a variety of clinical conditions, including cancer, renal failure, connective tissue disease, previous cardiac surgery and mediastinal radiation.^5^-^10^ Although the risk of developing CP is higher after tuberculous pericarditis,^11^ and ECP has been assumed to be a precursor to permanent constriction, the prevalence of tuberculous ECP is lower than that of idiopathic ECP.^3^

Although most patients with pericardial effusion respond well to medical therapy (especially of tuberculous aetiology),^12^ more than half of patients with ECP eventually need pericardiectomy due to persistent heart failure. The purpose of pericardiectomy (removal of the visceral pericardium) is to improve ventricular contractility; however, the early results and prognosis are still unknown in the surgical treatment of ECP.

## Methods

Twelve patients who underwent pericardiectomy and evacuation of pericardial fluid for ECP were included. These patients were among a total of 62 (19.2%) patients (50% male, median age 48 years, range 17–72) who underwent pericardiectomy for CP (15.8%) between November 2004 and March 2012. Patient registry information, medical records and hospital archives were systematically reviewed.

The underlying aetiology was idiopathic in five patients (41.6%), tuberculosis in four (33%), and malignancy in three (25%). Pre-operatively, all patients were receiving optimal medical therapy for congestive heart failure and non-steroidal anti-inflammatory drugs to decrease the pericardial effusion (Table 1).

**Table 1:** Baseline Characteristics

*Variable*	*Value (%)*
Clinical parameters	
NYHA class III or IV	9 (75.0)
Jugular venous distension	10 (83.3)
Ascites	9 (75.0)
Hepatomegaly	6 (50.0)
Hypertension	2 (16.7)
Diabetes	4 (33.3)
COPD	2 (16.7)
Creatinine > 1.5 µmol/l	4 (33.3)
Tobacco use	3 (25.0)
Pleural effusion	5 (41.7)
Echocardiography	
Ejection fraction (%)	60 (51.25–65)
Tricuspid insufficiency	5 (41.7)
Mitral insufficiency	4 (33.3)
Septal bounce	11 (91.6)
Plethora in IVC	10 (83.3)
Pericardial effusion	12 (100)
Biatrial dilatation	9 (75)

NYHA: New York Heart Association

Diagnosis was made primarily by echocardiography. ECP was diagnosed when widely accepted criteria for CP were met (thickening of parietal or visceral pericardium, asymmetric septal movement, plethored inferior vena cava, and variation in mitral or tricuspid inflow), accompanied by pericardial effusion. All patients met these echocardiographic criteria.

Right heart catheterisation (RHC) was performed in eight patients and equalisation of end-diastolic pressures within both ventricles was considered to be definitive for the presence of pericardial constriction. RHC was not performed in four patients; three who presented with acute cardiac tamponade underwent emergency surgery, and one patient had clear evidence of parietal pericardial thickening on transthoracic echocardiography.

Operative indications were advanced heart failure (i.e. NYHA class ≥ III) in eight patients (66.6%), acute cardiac tamponade in three (25%), and decreased effort capacity in one (8.3%) who had a recurrence of pericardial fluid accumulation. Overall, failure of therapeutic pericardiocentesis combined with medical treatment was the indication for surgery in 10 of the 12 patients (Table 2).

**Table 2:** Details Of Operations Performed

*Patient*	*Aetiology*	*Clinical status: indication for operation*	*Operation*	*Pericardiectomy*
1	ID	Advanced heart failure (NYHA IV) unresponsive to medical treatment	Elective	TP + epicardial fragmentation
2	TB	Heart failure (NYHA III)	Elective	TP + epicardial peeling
3	MG^†^	Cardiac tamponade – CPR	Emergency	PP + epicardial fragmentation
4	MG^‡^	Advanced heart failure (NYHA IV) unresponsive to medical treatment	Elective	TP
5	TB	Cardiac tamponade	Emergency	PP + epicardial peeling
6	ID	Heart failure (NHYA III)	Elective	TP + epicardial fragmentation
7	MG^§^	Advanced heart failure (NYHA IV) unresponsive to medical treatment	Elective	TP
8	TB	Heart failure (NYHA III)	Elective	TP + epicardial fragmentation
9	ID	Dyspnea, decreased effort capacity (NYHA II)	Elective	TP
10	ID	Cardiac tamponade	Emergency	PP + epicardial fragmentation
11	TB	Heart failure (NYHA III)	Elective	TP
12	ID	Heart failure (NYHA III)	Elective	TP

ID: idiopathic, TB: tuberculosis, MG: malignancy, NYHA: New York Heart Association, HF: heart failure, TP: total pericardiectomy, PP: partial pericardiectomy. ^†^Neoplastic cell invasion without definitive diagnosis, ^‡^Pericardial involvement of malignant mesothelioma (epitolid type), ^§^Pericardial involvement of high-grade diffuse β-type cell lymphoma.

Therapeutic pericardiocentesis was defined as percutaneous evacuation of the pericardial fluid to relieve cardiac functions. Pre-operatively, 10 patients had undergone at least one previous attempt at therapeutic pericardiocentesis. The procedure was routinely performed under echocardiographic guidance using a 5-F pigtail catheter inserted via the left infrasternal angle.

On admission, five patients underwent pericardiocentesis under transthoracic echocardiographic guidance and were referred for surgery because the constriction persisted despite evacuation of the fluid. For the remaining patients, pericardiocentesis was not re-attempted in five patients because the effusion had recurred after previous attempts (two attempts in four patients and one attempt in one patient), and it was not attempted at all in two patients due to multiple, dense septations observed on echocardiography. Pericardiocentesis was always combined with medical therapy, which consisted of oral administration of antiinflammatory agents. The use of corticosteroids was avoided.

During surgery, a median sternotomy was made in all patients. Beginning at the ascending aorta and anterolateral portion of the left ventricle, the anteroposterior extent of dissection was extended between the two phrenic nerves and included the superior vena cava–right atrium junction superiorly. The diaphragmatic surface and the inferior vena cava–right atrium junction defined the inferior extent of the excision. The atria and vena cavae were decorticated, if it could be performed without risk of haemorrhage.

For most patients, multiple sharp dissections were required to establish a true dissection plane. In some situations, when both layers were thickened without a clear boundary, some areas of fragmented and firm adhesions were left in situ unless ventricular motion was compromised. When ventricular motion was not improved, the thickened visceral pericardium was further divided into smaller fragments by electrocautery (epicardial fragmentation).

Total pericardiectomy was defined as wide excision of the anterior pericardium. Pericardiectomy was considered partial if both ventricles could not be decorticated because of dense adhesions.

## Statistical analysis

All statistical analysis was performed using SPSS version 15.0 software. Continuous variables were defined by the median ± interquartile ranges. Multiple measurements regarding the same parameter were compared using the Friedmann test. Values obtained pre- and postoperatively were compared using the Wilcoxon signed-ranks test. Survival was calculated using the lifetable method. A *p*-value of less than 0.05 was considered to be statistically significant.

## Results

Overall, four of 19 (21%) tuberculous patients, three of 10 (30%) malignancy patients, and five of 33 (15%) idiopathic patients had ECP. Nine patients had NYHA class > II symptoms, with the duration of heart failure ranging from two to 15 months (median nine months).

All except one patient were admitted for the first time, with duration of symptoms lasting between two and 12 months, with no identifiable cause of disease. The patient who had been admitted previously had undergone pericardiocentesis one month prior to the present admission. In this patient, histopathology revealed non-specific inflammation.

Of three patients with cancer, one had a six-year history of diffuse B-type cell lymphoma and one had a five-year history of both gastric lymphoma and pulmonary malignant mesothelioma. Both these patients had advanced heart failure and were unresponsive to medical treatment. An independent oncologist and lung specialist confirmed that expected lifespan was more than one year for both patients. The remaining patient had malignant cells on pericardial biopsy, indicative of metastatic disease of unknown origin. This patient had emergency surgery.

All tuberculous patients had a history of previous pulmonary involvement for a period of one to six years. All patients had documentation of completion of antituberculous treatment prior to admission. Acid-fast bacterial testing was negative pre-operatively on three separate occasions in all study patients.

Pre-operative echocardiography demonstrated a thickened parietal or visceral pericardium in 10 patients (83.3%), plethored inferior vena cava in 10 (83.3%), asymmetric septal movement in 11 (91.6%), and variation in mitral inflow in seven (58.3%). Overall, at least two criteria for CP were present, accompanied by varying amounts of pericardial effusion in all patients.

The amount of pericardial effusion was slightly higher on the right ventricular side (median 2.5 cm, interquartile range 2.1–3.3) than on the left ventricular side (median 2.3 cm, interquartile range 1.8–3.1 cm) and posterior wall (median 2.1 cm, interquartile range 1.2–2.3 cm). However, this difference was not found to be statistically significant (*p* = 0.076).

Five patients (41.7%) had tricuspid insufficiency, four (33.3%) had mitral insufficiency, and seven (58.8%) had bi-atrial dilatation. The left ventricular systolic function was within normal ranges in all patients (median ejection fraction 60%, interquartile range 51.25–65%).

The operation was elective in nine patients and emergency in three patients who presented with acute tamponade. The pericardial effusion consisted of free defibrinated blood in seven patients and was serofibrinated or loculated in the others. Samples of pericardial tissue and fluid were first sent for culture, histopathology and other testing prior to evacuation and volume measurement. Details of the operations performed are given in Table 2.

Acute haemorrhage and conversion to urgent cardiopulmonary bypass occurred in one patient. The bleeding was close to the posterolateral wall of the left ventricle and was treated with multiple pledgeted 4.0 polyprolene sutures. During peeling, minor haemorrhages occurring in the ventricular and right atrial wall were sutured using 5.0 polyprolene sutures. No coronary artery rupture or any additional complications were observed during the operation.

Pre-operatively, central venous pressure and pulmonary capillary wedge pressure were both high (median 16.5 mmHg, interquartile range 11.0–27.5; median 20.0 mmHg, interquartile range 16.0–23.5, respectively). Postoperatively, both showed a marked decrease. Central venous pressure decreased in seven patients, increased in two, and was unchanged in three (median 11.0 mmHg, interquartile range 10.0–16.0, *p* = 0.021). Pulmonary capillary wedge pressure decreased in eight patients and remained unchanged in four (median 15 mmHg, interquartile range 12.5–17.5, *p* = 0.011) (Table 3).

**Table 3:** Pre-Operative And Intra-Operative Data

				*Echocardiography*				*Catheterisation*				*Operation*				
*Patient*	*Aetiology*	*Age*	*Presentation*	*CP*	*ELVS (cm)*	*ERVS (cm)*	*EPWS (cm)*	*mRAP (mmHg)*	*RVEDP (mmHg)*	*LVEDP (mmHg)*	*mPAP (mmHg)*	*CVP 1 (mmHg)*	*PCWP 1 (mmHg)*	*Fluid removed (ml)*	*CVP 2 (mmHg)*	*PCWP 2 (mmHg)*
1	ID	48	HF	+	2.5	2.6	2.8	25	30	30	22	11	18	500	7	14
2	HF	47	HF	+	1.2	1.8	1.5	28	28	30	35	30	22	1400	16	18
3	MG	59	CT	+	2.8	3.6	2.2	-	-	-	-	10	24	1000	10	20
4	MG	48	HF	+	4.1	3.5	2.4	26	30	29	29	9	24	1000	11	16
5	TB	72	CT	+	3.2	2.4	4.1	-	-	-	-	11	22	1500	11	14
6	ID	67	HF	+	1.8	2.6	2.1	28	25	23	35	17	16	500	9	16
7	MG	50	HF	+	1.5	3.6	2.2	10	10	12	25	20	16	750	11	16
8	TB	34	HF	+	2.8	3.0	2.1	-	-	-	-	22	24	1500	16	14
9	ID	29	Dyspnoea	+	1.8	1.9	1.2	20	12	12	25	21	12	250	21	12
10	ID	49	CT	+	2.1	2.4	1.8	-	-	-	-	28	16	1200	16	10
11	TB	17	HF	+	3.5	2.2	1.0	25	12	15	22	15	12	600	10	12
12	ID	42	HF	+	2.2	2.1	1.2	21	23	23	18	16	22	400	19	18

ID: idiopathic, TB: tuberculosis, MG: malignancy, NYHA: New York Heart Association, CT: cardiac tamponade, CP: constrictive pericarditis, ELVS: effusion along left ventricle side, ERVS: effusion along right ventricle side, EPWS: effusion along posterior wall side, mRAP: mean right atrial pressure, RVEDP: right ventricular end-diastolic pressure, LVEDP: left ventricular end-diastolic pressure, mPAP: mean pulmonary artery pressure, CVP 1: central venous pressure before operation, CVP 2: central venous pressure after operation, PCWP 1: pulmonary capillary wedge pressure before operation, PCWP 2: pulmonary capillary wedge pressure after operation. CVP and PCWP measurements were performed using a Swan-Ganz catheter introduced via the internal jugular vein.

There was no in-hospital mortality. Respiratory distress was the most common postoperative complication, followed by low-cardiac output syndrome and renal failure. Two patients underwent re-operation for bleeding, although no particular site of bleeding was found in either one (Table 4).

**Table 4:** Operative And Postoperative Parameters

*Operative parameter*	*Value (%)*
Complete pericardiectomy	9 (75.0)
Time of operation (min)*	90 (90–120)
Ventilation > 8 hours	4 (33.3)
24 hours bleeding (ml)*	525 (362.5–837.5)
Re-operation for bleeding	2 (16.6)
Fluid removed (ml)*	875 (500–1350)
Transfusion (1 unit of ES)	5 (41.7)
Arrhythmia	3 (25.0)
LCOS	7 (58.3)
ICU stay > 3 days	7 (58.3)
ICU stay > 7 days	3 (25)
Peri-operative mortality	0 (0)

ES: erythrocyte suspension; LCOS: low-cardiac output syndrome; ICU: intensive care unit. *Data represented as medians with interquartile ranges.

**Fig. 1.**
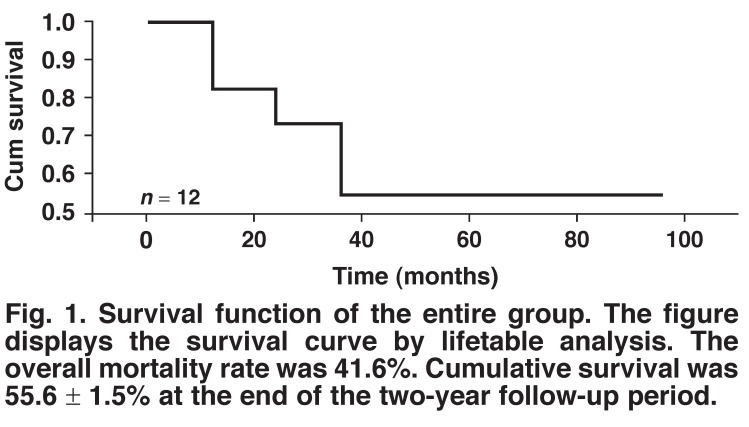
Survival function of the entire group. The figure displays the survival curve by lifetable analysis. The overall mortality rate was 41.6%. Cumulative survival was 55.6 ± 1.5% at the end of the two-year follow-up period.

The follow-up period ranged from three months to nine years (median three years). Overall, five patients (41.6%) died from various causes. The three cancer patients died from disease progression within two years postoperatively, whereas all five tuberculous patients survived to the end of the follow-up period. Cumulative survival was 55.6 ± 1.5% at the end of the two-year follow-up period (when the last death occurred). Seven patients survived with a median follow up of five years (range nine months to eight years) postoperatively (Table 5, Fig. 1).

**Table 5:** Results Of Pericardial Tissue Biopsy And Follow-Up Data

*Patient*	*Aetiology*	*Date of operation*	*Intensive care unit stay*	*Pericardial biopsy*	*Follow up (months)*	*Outcome*
1	ID	2004	8 days, LCOS, RF, RDS	Non-specific inflammation	4.04	Death from pneumonia + sepsis
2	TB	2004	1 day, uneventful	Granulomatous inflammation	95.0	NYHA class I
3	MG	2005	7 days, LCOS, RF, RDS	Neoplastic involvement^†^	2.9	Death from disease progression
4	MG	2005	2 days, uneventful	Neoplastic involvement^‡^	19.7	Death from disease progression
5	TB	2006	8 days, re-operation for bleeding, RF, RD	Granulomatous inflammation	79.9	NYHA class III
6	ID	2006	6 days, re-operation for bleeding, RF, RD	Non-specific inflammation	25.7	Death from advanced HF
7	MG	2007	2 days, uneventful	Neoplastic involvement^§^	25.6	Death from disease progression
8	TB	2007	8 days, LCOS, RD	Non-specific inflammation	66.4	NYHA class II
9	ID	2007	1 day, uneventful	Non-specific inflammation	62.5	NYHA class I
10	ID	2008	5 days, low-dose inotrope	Non-specific inflammation	51.3	NYHA class I
11	TB	2008	2 days, uneventful	Granulomatous inflammation	48.2	NYHA class II
12	ID	2012	3 days, low-dose inotrope	Non-specific inflammation	8.9	NYHA class II

ID: idiopathic, TB: tuberculous, MG: malignancy, LCOS: low-cardiac output syndrome, RF: renal failure, RD: respiratory distress, NYHA: New York Heart Association, HF: heart failure. ^†^Neoplastic cell invasion without definitive diagnosis, ^‡^Pericardial involvement of malignant mesothelioma (epitolid type), ^§^Pericardial involvement of high-grade diffuse B-type cell lymphoma.

## Discussion

Our study emphasises the clinical importance of ECP. Survival after pericardiectomy for ECP was lower than previously reported.^13^,^14^ Although ECP has been observed in only a minority of pericarditis patients, we found that it represented a higher proportion in pericardiectomy patients. Moreover, we found that surgery for ECP was associated with substantial morbidity, without early mortality. Various complications occurred in 41.6% (*n* = 5) of patients, with more than half the patients having a prolonged intensive care unit stay.

Total pericardiectomy, which we defined as peeling the parietal pericardium between the phrenic nerves anteriorly and peeling its diaphragmatic surface inferiorly, was achieved in nine patients. Pericardiectomy was limited (partial) in three patients in whom the decortication border could not satisfactorily be extended through the lateral aspect of the left ventricle because of dense calcific adhesions. Also, fragmented areas of the epicardium had to be left without peeling and could not be included within the decortication border in five patients.

Although pre-operative ejection fractions of the patients were within normal ranges, postoperative LCOS was observed in seven patients. High rates of LCOS may partly have been due to structural alterations within the ventricle myocardium, which had developed due to long-lasting constriction during the chronic disease period. However, inadequate decortication of the left ventricle was the most probable reason for the development of high rates of LCOS, but this could not be totally proven.

The initial approach should include a thorough clinical evaluation, with pericardiocentesis under echocardiographic guidance, and heart catheterisation available. The existence of persistent high intracardiac pressures, despite evacuation of the effusion, is essential for an accurate diagnosis; ECP was present in only 6.8% of patients undergoing pericardiocentesis in Sagrista-Sauleda and co-workers’ study.^2^ Effective pericardiocentesis is of therapeutic importance, especially in the setting of pericardial tamponade.

ECP has increasingly become a subject of intense research. Hancock was the first to describe the condition as a particular form of pericardial constriction, which persists despite evacuation of the compressive pericardial effusion.^1^ Although its prevalence ranged between 2.4 and 14.8%,^3^ Cameron reported the proportion of ECP to be as high as 24% in patients requiring pericardiectomy.^4^

The first prospective study by Sagrista-Sauleda and co-workers identified 15 ECP patients among 190 with tamponade over a period of 16 years.^2^ In this study, an accurate diagnosis of ECP was made with combined pericardiocentesis and cardiac catheterisation. The aetiological spectrum was similar to that reported in previous studies, with a predominance of idiopathic cases. Other less-frequent causes included post-cardiac surgery, tuberculosis, post-radiation and neoplasia. Seven of 15 (46%) patients underwent pericardiectomy within four months after pericardiocentesis and two patients died in the early postoperative period.

Patients with cancer were found to have a high mortality and low pericardiectomy rate, whereas patients with idiopathic causes of ECP had a low mortality but high pericardiectomy rate. Also, four of the six survivors ultimately required pericardiectomy.^2^ We concluded that the development of persistent constriction is frequent in ECP and extensive epicardiectomy is the procedure of choice in patients with persistent heart failure.

A recent systematic review by Ntsekhe *et al.*^3^ identified a pooled prevalence of 4.5% by applying a random-effects model. The aetiological spectrum was similar to that of previous ECP and CP series, although neoplastic and traumatic cases were excluded from the analysis; 26 ECP patients were identified among 642 subjects derived from five observational studies. The series reported by Sagrista-Sauleda *et al.* was also included in this review with its 11 non-neoplastic patients.^2^

In our study, pre-operative echocardiography of all ECP patients met the widely accepted criteria for ECP. Cardiac catheterisation was performed without concomitant pericardiocentesis and revealed equalisation of interventricular pressures in eight patients. However, we lacked intra-operative haemodynamic data regarding the effect of the presence and evacuation of pericardial fluid. Histopathology of the pericardial fluid and tissue were consistent with the underlying disease (Table 5). Five patients died during follow up; two of these deaths occurred within the first four months.

Similar to previous reports, our patients were unresponsive to pericardiocentesis and subsequent aggressive medical therapy. Although early mortality did not occur, the majority of our patients had a complicated postoperative course. ECP has a relatively long duration of symptoms and failure of repeated pericardiocentesis attempts; therefore, echocardiography should not be seen as a misleading diagnostic tool. The presence of signs consistent with ECP should prompt early surgical intervention, especially in patients with known underlying disease, such as cancer or tuberculosis.

The prognosis depends on the underlying disease in cancer patients, whereas patients with idiopathic ECP may respond to subsequent medical treatment after pericardiocentesis. Patients should be closely observed for the recurrence of symptoms; re-accumulation of fluid should be considered as indicative of disease persistence.

## Conclusion

Pericardiectomy for ECP was effective, in terms of our early results, in patients unresponsive to medical therapy. Long-term survival depends on the underlying disease. The decision to delay or not to delay surgery in specific aetiological subgroups should be one of the main considerations for future studies on ECP.
